# Molecular evidence provides new insights into the evolutionary origin of an ancient traditional Chinese medicine, the domesticated “Baizhi”

**DOI:** 10.3389/fpls.2024.1388586

**Published:** 2024-05-08

**Authors:** Yingyu Lin, Gang Yao, Chunxiu Huang, Zhi Chao, Enwei Tian

**Affiliations:** ^1^ School of Traditional Chinese Medicine, Southern Medical University, Guangzhou, China; ^2^ College of Forestry and Landscape Architecture, South China Agricultural University, Guangzhou, China; ^3^ Guangdong Provincial Key Laboratory of Chinese Medicine Pharmaceutics, Southern Medical University, Guangzhou, China; ^4^ Guangdong Provincial Engineering Laboratory of Chinese Medicine Preparation Technology, Southern Medical University, Guangzhou, China

**Keywords:** Baizhi, original plant, nrITS, plastid genome, phylogenetic relationships

## Abstract

**Introduction:**

“Baizhi” is a famous herbal medicine in China, and it includes four landraces named as ‘Hangbaizhi’, ‘Chuanbaizhi’, ‘Qibaizhi’, and ‘Yubaizhi’. Long-term artificial selection had caused serious degradation of these germplasms. Determining the wild progenitor of the landraces would be benefit for their breed improvements. Previous studies have suggested *Angelica dahurica* var. *dahurica*, *A. dahurica* var. *formosana*, or *A. porphyrocaulis* as potential candidates, but the conclusion remains uncertain, and their phylogenetic relationships are still in controversy.

**Methods:**

In this study, the genetic variation and phylogenetic analyses of these species and four landraces were conducted on the basis of both the nrITS and plastome datasets.

**Results:**

Genetic variation analysis showed that all 8 population of four landraces shared only one ITS haplotype, meanwhile extremely low variation occurred within 6 population at plastid genome level. Both datasets supported the four landraces might be originated from a single wild germplasm. Phylogenetic analyses with both datasets revealed largely consistent topology using Bayesian inference and Maximum likelihood methods. Samples of the four landraces and all wild *A. dahurica* var. *dahurica* formed a highly supported monophyletic clade, and then sister to the monophyly clade comprised by samples of *A. porphyrocaulis*, while four landraces were clustered into one clade, which further clustered with a mixed branches of *A. porphyrocaulis* and *A. dahurica* var. *dahurica* to form sister branches for plastid genomes. Furthermore, the monophyletic *A. dahurica* var. *formosana* was far distant from the *A. dahurica* var. *dahurica*-“Baizhi” clade in *Angelica* phylogeny. Such inferences was also supported by the evolutionary patterns of nrITS haplotype network and K2P genetic distances. The outcomes indicated *A. dahurica* var. *dahurica* is most likely the original plant of “Baizhi”.

**Discussion:**

Considering of phylogenetic inference and evolutionary history, the species-level status of *A. dahurica* var. *formosana* should be accepted, and the taxonomic level and phylgenetic position of *A. porphyrocaulis* should be further confirmed. This study preliminarily determined the wild progenitor of “Baizhi” and clarified the phylogenetic relationships among *A. dahurica* var. *dahurica*, *A. dahurica* var. *formosana* and *A. porphyrocaulis*, which will provide scientific guidance for wild resources protections and improvement of “Baizhi”.

## 1 Introduction

Angelicae Dahuricae Radix (Chinese name “Baizhi”), which was firstly recorded in Sheng Nong’s herbal classic published in the Eastern Han Dynasty (at least 1,800 years ago), is a famous traditional Chinese medicine (Wang et al., 2020). Previous studies have revealed the presence of primary active components in “Baizhi,” such as coumarins, volatile oils, and flavonoids (Tabanca et al., 2014; Cao et al., 2017; Zhao et al., 2022). “Baizhi” has the effects of anti-inflammatory (Lee et al., 2011; Yang et al., 2015; Wei et al., 2016), antioxidant (Wang et al., 2017; Liang et al., 2018; Shu et al., 2020), antimicrobial (Kwon et al., 1997; Yang et al., 2020), etc., and it has been widely used to eliminate wind cold and dampness, expel pus, relieve pain, and decrease blood glucose levels (The national pharmacopoeia commission, 2020). In addition to medical uses, “Baizhi” was also widely used as a raw material of health products, cosmetics, and flavorants (Liu et al., 2020), which is in great demand in China, Korea, and Japan (Noh et al., 2018).

The modern medicinal “Baizhi” includes four different landraces, which were named according to their localities of cultivation in China, viz., ‘Hangbaizhi’ cultivated in Zhejiang province, ‘Chuanbaizhi’ cultivated in Sichuan province, ‘Yubaizhi’ cultivated in Henan province, and ‘Qibaizhi’ cultivated in Hebei province. These four landraces have been cultivated (seed propagated) for hundreds to thousands of years (Zhong, 1956; Wang et al., 2001d; Wang and Jia, 2004; Niu et al., 2018; Wang et al., 2020; Zheng et al., 2020; Zhu, 2023). Long-term cultivation and selection often narrow the genetic background, resulting in the loss of genetic diversity (Kassa et al., 2012), and may further lead to the degeneration of the cultivated germplasm of plants, such as declined resistance to disease and insects and decreased quality and yield (Ma et al., 2009). In fact, previous studies have shown that during the cultivation processes, the four landraces of “Baizhi” were susceptible to attack by diseases and pests in different growth periods, accompanied by various problems such as rotten roots, premature bolting and flowering, and severe lignification, which affected the quality of medicinal material and thus caused it to be unqualified for official use (Luo et al., 1996; Xue et al., 2009). As revealed in our previous study on the basis of 12 polymorphic microsatellites, the genetic diversity among the four cultivated landraces was relatively low. The genetic diversity of ‘Qibaizhi’ and ‘Yubaizhi’ landraces (*H*o = 0.301) was lower than that of ‘Hangbaizhi’ and ‘Chuanbaizhi’ landraces (*H*o = 0.340) (Liu et al., 2020; Huang et al., 2022), providing further evidence of the reduced adaptability of the landraces due to the long-term artificial selection. Generally, wild medicinal resources possess great genetic variations and beneficial genes before domestication and artificial selection, providing a reservoir of genetic variation for improving the adaptability, yield, and quality of cultivated medicinal varieties (Shams et al., 2020). However, up to date, the wild original plant of the four landraces has not been clarified yet, which seriously limits the improvement and breeding advances of new varieties of “Baizhi”.

During the past decades, researchers have made great efforts to address the issue on the original plant of the four cultivated landraces. Most researchers thought the wild original plant of the cultivated landraces might fall into three taxa of two species in the genus *Angelica* (Apiaceae), viz., *Angelica dahurica* var. *dahurica* (Fischer ex Hoffmann) Bentham & J. D. Hooker ex Franchet & Savatier, *A. dahurica* var. *formosana* (H. de Boissieu) Yen, and *A. porphyrocaulis* Nakai & Kitagawa (Wang et al., 2001a; Huang, 2004; Wang and Jia, 2004; Wang et al., 2020). Based on analyses of both macro- and micro-morphology as well as the cultivation history, Wang et al. (2001a; 2001b; 2001c; 2001d) had suggested that there were no evident differences among different landraces of “Baizhi,” and they further revealed that the four landraces might originate from the wild population of *A. dahurica* var. *formosana*, which is endemic to Taiwan, China. Wang et al. (2001d) also suggested that *A. porphyrocaulis* should be treated as a variety of *A. dahurica*, viz. *A. dahurica* var. *porphyrocaulis*. Huang (2004) integrated the studies from the comparisons of morphological characters, chromosome karyotypes, chemical compositions, and genetic variations between cultivated populations of these four landraces and wild species of *Angelica*, further confirming that the wild germplasm resources of “Baizhi” might derive from *A. dahurica* var. *formosana*. Meanwhile, relevant studies also revealed that the three taxa might be closely related and thus shared a common ancestor. On the other hand, through sorting out the historical records of material medica, Wang and Jia (2004) thought that the historically cultivated “Baizhi” originated from *A. dahurica* or its closely related species. In addition, by consulting the ancient traditional Chinese medicinal books and modern relevant literature, On the other hand, Wang et al. (2020) even suggested that the four landraces of “Baizhi” might originate from two different taxa, viz., ‘Qibaizhi’ and ‘Yubaizhi’ originated from *A. dahurica* var. *dahurica*, yet ‘Hangbaizhi’ and ‘Chuanbaizhi’ originated from *A. dahurica* var. *formosana*. The description in *Flora of China* is quite different from the previous points of view. It is recorded that the landraces of ‘Qibaizhi’ and ‘Yubaizhi’ and the landraces of ‘Hangbaizhi’ and ‘Chuanbaizhi’ belong to two different cultivars of *A. dahurica*, named *A. dahurica* cv. ‘Qibaizhi’ and *A. dahurica* cv. ‘Hangbaizhi’, respectively. However, no wild populations of these two taxa have been found in the field to date (Pan and Watson, 2005).

Although lots of efforts have been made in tracing to the wild sources of the four landraces of “Baizhi,” there is still a big dispute to date as mentioned above. Briefly, the focuses of controversy are as follows: (1) Do the four landraces need to be classified in taxonomy? If not, do they originate from the same wild germplasm resources, *A. dahurica* var. *dahurica* or *A. dahurica* var. *formosana*? If so, do the ‘Qibaizhi’ and ‘Yubaizhi’ landraces originate from *A. dahurica* var. *dahurica*, whereas the ‘Hangbaizhi’ and ‘Chuanbaizhi’ landraces originate from *A. dahurica* var. *formosana*? (2) Should the related species, *A. porphyrocaulis*, be merged into *A. dahurica* or treated as a separate species or subspecies?

In this study, the samples of “Baizhi” landraces (‘Hangbaizhi’, ‘Chuanbaizhi’, ‘Qibaizhi’, and ‘Yubaizhi’) and three taxa of two species in the genus *Angelica* (Apiaceae) taxa, viz., *A. dahurica* var. *dahurica*, *A. dahurica* var. *formosana*, and *A. porphyrocaulis*, were collected in China, covering their major distribution areas. The nrITS and plastid genome have been demonstrated effectively in molecular phylogeny and genetic variation studies of the *Angelica* (Apiaceae) (Yuan et al., 2015; Wang et al., 2021). Here, we used population-level datasets of nrITS and plastid genome to conduct genetic variation and phylogenetic analyses, aiming to (1) elucidate whether the landraces were derived from one wild germplasm; (2) infer the wild original plant of these landraces; and (3) clarify the taxonomic position of the original plant and its phylogenetic relationship with related *Angelica* species. The results are expected to provide new insight into the evolutionary origin of the “Baizhi” landraces and clarify the protected and utilized objects, further advancing the genetic improvement and breeding of “Baizhi”.

## 2 Materials and methods

### 2.1 Plant material collection and DNA extraction

**Figure 1:**
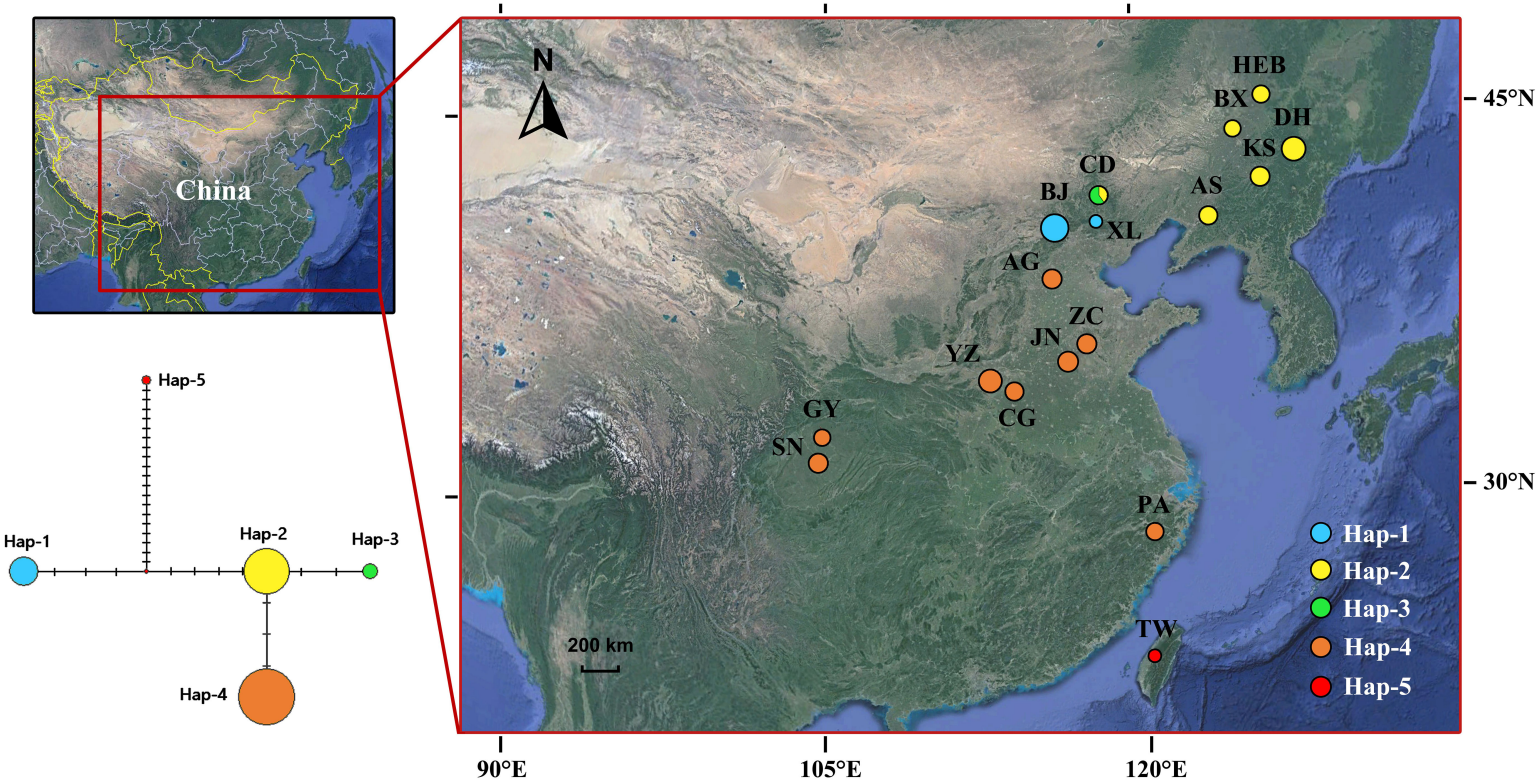
ITS Haplotypes geographical distributions and network of four landraces and related *Angelica* species.

A total of 86 individuals representing all the four landraces of “Baizhi” were collected from the main producing areas (including Zhejiang, Sichuan, Hebei, and Henan provinces) of the medicinal plants in China. Additionally, 63, 1, and 22 individuals of the three taxa of *Angelica*, viz. *A. dahurica* var. *dahurica*, *A. dahurica* var. *formosana*, and *A. porphyrocaulis*, were also collected, respectively (**Table 1**; **Figure 1**). In total, 172 individuals were used for nrITS analysis. Meanwhile, 19 specimens were used for plastid genome analysis (1~4 individuals for each species or landrace). The fresh leaves of these samples were dried with silica gel, except the specimen of *A. dahurica* var. *formosana* (specimen voucher no.: 00674195) which was stored in the herbarium of South China National Botanical Garden, Chinese Academy of Science. Sampling information of all samples is listed in **Table 1**. Total genomic DNA were extracted from the dried leaves using the CTAB method (Yang et al., 2014). The concentration and integrity of the genomic DNA were assessed using a NanoDrop 1000 UV/Vis spectrophotometer (Thermo Scientific, Wilmington, DE, USA) and 1.5% agarose gel electrophoresis, respectively.

**Table 1:** Sampling information of *Angelica* species and four landraces.

Species (landraces)	Locations (population ID)	Growth types	Latitude, longitude	Sample size for nrITS analysis	Sample size for plastid genome analysis
Angelica porphyrocaulis	Beijing (BJ)	Wild	39°58′47″ N, 115°25′40″ E	21	1
	Xinglong, Hebei (XL)	Wild	40°34′56″ N, 117°30′19″ E	1	/
	Chengde, Hebei (CD)	Wild	40°40′12″ N, 117°40′12″ E	10	1
	Benxi, Liaoning (BX)	Wild	44°22′47″ N, 124°57′58″ E	8	1
	Anshan, Liaoning (AS)	Wild	41°00′55″ N, 123°08′05″ E	10	1
	Tonghua, Jilin (KS)	Wild	42°25′49″ N, 126°06′36″ E	11	1
Angelica dahurica var. dahurica	Dunhua, Jilin (DH)	Wild	43°34′14″ N, 128°00′50″ E	15	/
	Haerbin, Heilongjiang (HEB)	Wild	45°42′24″ N, 126°38′37″ E	9	1
‘Hangbaizhi’ landrace	Panan, Zhejiang (PA)	Cultivated	28°57′05″ N, 120°28′05″ E	9	2
‘Chuanbaizhi’ landrace	Guangyuan, Sichuan (GY)	Cultivated	31°56′38″ N, 105°38′39″ E	8	2
	Suining, Sichuan (SN)	Cultivated	30°34′09″ N, 105°34′49″ E	11	2
‘Qibaizhi’ landrace	Anguo, Hebei (AG)	Cultivated	38°25′11″ N, 115°19′37″ E	11	2
	Jining, Shandong (JN)	Cultivated	35°23′11″ N, 116°40′31″ E	12	/
	Zoucheng, Shandong (ZC)	Cultivated	35°24′36″ N, 116°53′47″ E	11	/
‘Yubaizhi’ landrace	Changge, Henan (CG)	Cultivated	34°11′30″ N, 113°53′45″ E	10	2
	Yuzhou, Henan (YZ)	Cultivated	34°12′01″ N, 113°34′32″ E	14	2
Angelica dahurica var. formosana	Nantou, Taiwan (TW)	Wild	/	1	1
Total				172	19

“/” indicates no samples were used for analysis or no corresponding information.

### 2.2 Nuclear ribosomal ITS sequence analysis

The universal primers of the nuclear ribosomal internal transcribed spacer (ITS; ITS4: 5′-TCCTCCGCTTATTGATATGC-3′; ITS5: 5′-GGAAGTAAAAGTCGTAACAAGG-3′ (White et al., 1990) were used to amplify the ITS sequences of 172 individuals of four landraces of ‘Baizhi’ and the three taxa, viz., *A. dahurica* var. *dahurica*, *A. dahurica* var. *formosana*, and *A*. *porphyrocaulis*. Polymerase chain reaction (PCR) was performed in a volume of 20 μL consisting of 20 ng genomic DNA, 0.2 mM each dNTP, 0.4 μM each primer, 10× PCR buffer (Mg^2+^ free), 2.5 mM Mg^2+^, and 1 unit Taq DNA polymerase (Takara, Dalian, China) with the following procedure: initial denaturation at 94°C for 4 min, 35 cycles of denaturation at 94°C for 40 s, annealing at 55°C for 60 s, extension at 72°C for 45 s and a final extension of 72°C for 8 min. The purified PCR products were sent to Beijing Ruibiotech Co., Ltd. (Beijing, China) for Sanger sequencing. All obtained sequences excluding primer regions were edited and assembled using a program of SeqMan supplemented in DNASTAR v11.1.0.54 assisted with manual correction (Burland, 1999). Multiple-sequence alignments were carried out by Muscle implemented in MEGA 5.0 (Tamura et al., 2011) and then modified artificially.

Polymorphism indices for each taxon (or landraces) were calculated using the software DnaSP v5.0 (Librado and Rozas, 2009). Polymorphism indices included the number of haplotypes (*K*), number of segregating sites (*S*), nucleotide diversity (Pi), and haplotype diversity (*H*d). Pairwise intra- and interspecific genetic distances for ITS were analyzed based on the Kimura two-parameter (K2P) model through MEGA v5.0 (Tamura et al., 2011).

To understand the relationships between the four landraces and the two wild species (including three taxa: *A. dahurica* var. *dahurica*, *A. dahurica* var *formosana*, and *A. porphyrocaulis*), phylogenetic inference was carried out using both Bayesian inference (BI) and maximum likelihood (ML) methods implemented in PhyloSuite v1.2.2 (Zhang et al., 2020). A 52-ITS-sequence matrix that consisted of 44 sequences (24 *Angelica* species) downloaded from NCBI (https://www.ncbi.nlm.nih.gov/) and 8 sequences from 5 haplotypes (including the above three taxa and four cultivated landraces) sequenced by this study was used for phylogenetic analyses (Accession nos. are shown in **Supplementary Table 1**). Two closely related species of Apioideae (*Coriandrum sativum* Accession no.: HQ377205 and *Foeniculum vulgare* Accession no.: AY551289, HQ377213) were selected as the outgroups. The best-fit substitution models (SYM+G) were determined using the jModelTest v2.1.10 under the standard of the Akaike information criterion (AIC) for BI and ML inference (Posada, 2008). For the BI analysis, five chains of 500,000 generations were conducted for the Markov chain Monte Carlo (MCMC) analysis with trees sampled every 1,000 generations. The first 25% of the sampled trees were discarded as burn-in, and the remaining trees were used to build a 50% majority-rule consensus tree. For the ML analysis, branch support was assessed with 1,000 bootstrap replications. The consensus tree was checked and edited with FigTree v1.4.3 (http://tree.bio.ed.ac.uk/software/figtree/). For further comparison, relationships among five haplotypes in the four landraces and the three taxa (172 samples in total) were also estimated using a statistical parsimony network approach in TCS 1.21 (Clement et al., 2000) with loops in the network resolved (Crandall and Templeton, 1993).

### 2.3 Plastid genome sequence analysis

100~200 ng high-quality genomic DNA of the 19 samples (**Table 1**) was respectively cut into 500-bp contigs with the Covaris S220 Focused-ultrasonicator for the paired-end library construction according to the manufacturer’s manual (Illumina Inc., San Diego). The library was constructed using the NEBNext Ultra II DNA library Prep Kit (Illumina). DNA paired-end sequencing of 150 bp was run by BGISEQ-500 (BGI, Shenzhen, China). The raw reads obtained were trimmed for quality control using NGS QC Toolkit with default settings (Patel and Jain, 2012). Then, the trimmed sequences were assembled into contigs using *de novo* assemble in GetOrganelle v.1.7.0 (Jin et al., 2020) and SPAdes (Bankevich et al., 2012) with the default parameters. The published plastid genome of *A. dahurica* var. *dahurica* (Accession no.: NC029392) was set as reference to check the errors and ambiguities of the assembled contigs using Geneious v8.1 (Kearse et al., 2012). The plastid genome was then annotated using Plastid Genome Annotator (PGA) software (Qu et al., 2019) and Dual Organellar GenoMe Annotator (Wyman et al., 2004) and checked manually. The start and stop codon exon and intron boundaries of coding sequences (CDs) of the plastid genome were manually adjusted *via* Geneious v8.1. The boundaries of the large single copy region (LSC), small single copy region (SSC), and inverted repeats (IRs) for *Angelica* species (or landraces) plastid genomes were also verified by the Find Repeats in Geneious v8.1. The tRNA genes were confirmed using the tRNAscan-SE v.2.0.3 (Schattner et al., 2005) and ARAGORN v1.2.38 (Laslett and Canback, 2004) with the default parameters. The OGDRAW software v1.3.1 was used to draw circular plastid genome maps (Lohse et al., 2007). The 19 plastid genome sequences were deposited into GenBank (Accession no. were shown in **Supplementary Table 2**).

All the plastid genome sequences were aligned with MAFFT v7.037 (Kuraku et al., 2013) and then adjusted manually by MEGA v5.0. Pairwise intraspecific and interspecific genetic distances for the four landraces and two related species were analyzed basing on the K2P model through MEGA 5.0. All the 19 samples were used to detect the variation within the four landraces and two related species through mVISTA online software (https://genome.lbl.gov/vista/mvista/submit.shtml) with *A. dahurica* var. *dahurica* (HEB18) as reference. Sliding window analysis was conducted using DnaSP v5.0 to analyze nucleotide diversity (Pi). There were 12 samples from four cultivated landraces that were also used to performed mVISTA analysis and sliding window analysis separately with the ‘Hangbaizhi’ landrace (PA29) as reference. Phylogenetic inference was conducted with a dataset of the complete plastid genomes from four landraces (12 sequences) and two related species [*A. dahurica* var. *dahurica* (5 sequences), *A. dahurica* var *formosana* (1 sequence), and *A. porphyrocaulis* (1 sequence)] and other species (28 species, 45 sequences) in the genus of *Angelica* (Apiaceae) downloaded from GenBank (**Supplementary Table 2**). The species *Foeniculum vulgare* (Accession numbers: KR011054) was used as outgroup. We used the identical methods (ML and BI) to reconstruct the phylogenetic tree. The best-fitting substitution models (TVM+G) were selected based on the AIC using jModelTest v2.1.10 (Posada, 2008). The ML phylogeny was performed using RAxML v8.12 software (Stamatakis, 2014). The bootstrap support was calculated with 1,000 replications. The BI inference referred to the above methods in phylogenetic analysis of ITS datasets. The phylogenetic trees were plotted and edited in FigTree v1.4.3 (http://tree.bio.ed.ac.uk/software/figtree/).

## 3 Results

### 3.1 Characteristics and genetic variations of nrITS and plastid genome among the four landraces and two related *Angelica* species

The aligned matrix of the nrITS sequence contained 633 base pairs (bp), which included 31 variation sites (including 12 parsimony informative sites and 19 singleton variable sites). Five haplotypes were identified among the 172 ITS sequences. Only one haplotype was held in *A. porphyrocaulis* (Hap 1), *A. dahurica* var. *formosana* (Hap 5), and four landraces of “Baizhi” (Hap 4), respectively, whereas *A. dahurica* var. *dahurica* has two haplotypes (Hap 2 and Hap 3) over the six sampling populations (**Table 1**; **Figure 1**), and it has the highest haplotype diversity (*H*d = 0.175) and nucleotide diversity (Pi = 0.00056) (**Table 2**). Among the five haplotypes, Hap 4 (four landraces) is the most widespread, which distributed in both the central and southern regions of China (eight cultivated populations), whereas Hap 2 and Hap 3 (*A. dahurica* var. *dahurica*) distributed only in northern and northeastern China (six wild populations). Hap 1 (*A. porphyrocaulis*) covered the transitional zones (Beijing and Hebei populations). Hap 5 (*A. dahurica* var. *formosana*) only harbored in Taiwan island of China (**Figure 1**).

**Table 2:** The number of haplotypes and polymorphism for the four landraces and two related *Angelica* species.

Species/landraces	Number of haplotypes (*K*)	Number of polymorphic sites (*S*)	nucleotide diversity (Pi)	haplotype diversity (*H*d)	Haplotype name (number of specimens)
Angelica porphyrocaulis	1	0	0	0	Hap 1 (22)
Angelica dahurica var. dahurica	2	2	0.00056	0.175	Hap 2 (57), Hap 3 (6)
Four landraces	1	0	0	0	Hap 4 (86)
Angelica dahurica var. formosana	1	/	/	/	Hap 5 (1)

“/” indicates no corresponding result.

**Figure 2:**
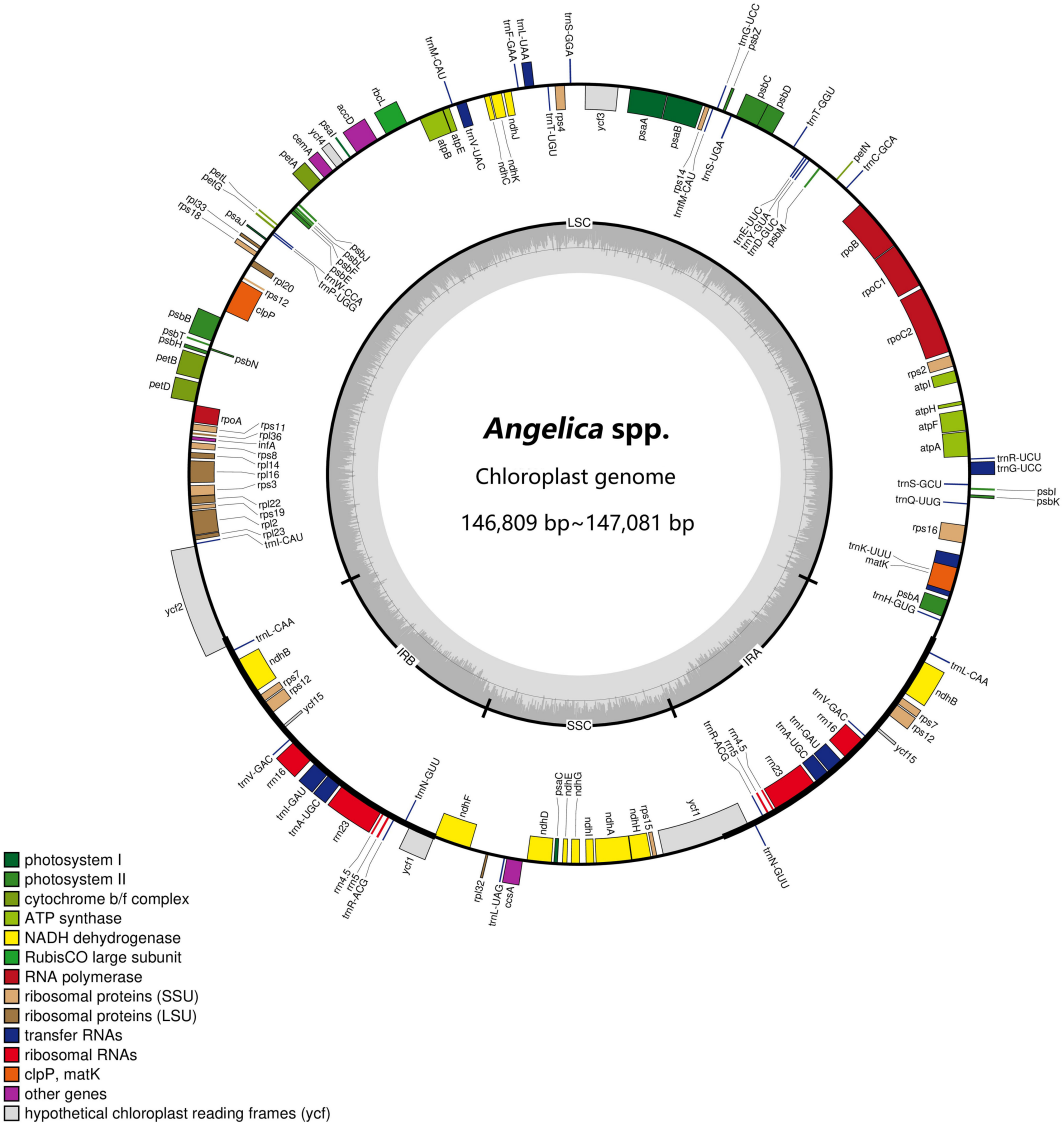
Plastid genome map of the four landraces and two related *Angelica* species. Genes inside the circle are transcribed clockwise, and those outside are counterclockwise. Genes of different functions are color-coded. The darker gray in the inner circle shows the GC content, whereas the lighter gray shows the AT content.

We obtained 19 complete plastid genome sequences of the two *Angelica* species and four landraces. The characteristics of these plastid genomes are displayed in **Table 3**. The plastid genomes ranged from 146,809 bp [‘Hangbaizhi’ landrace, (PA29)] to 147,081 bp [*A. dahurica* var. *dahurica* (CD25)] in length, displaying a typical quadripartite circular structure with a pair of IR region (size: 17,814 bp~18,078 bp) separated by LSC (size: 93,314 bp~93,602 bp) and SSC region (size: 17,629 bp~17,672 bp) for the four landraces and two related *Angelica* species (**Figure 2**; **Table 3**). The GC content of the complete plastid genomes ranged from 37.51% to 37.54%, and that of LSC region ranged from 35.89% to 35.92%. *A. dahurica* var. *formosana* was different in GC content of the SSC and IR regions (44.84% in the IR region and 30.95% in the SSC region) compared with the other two taxa and four landraces (44.95%~44.98% in the IR region and 31.05%~31.09% in the SSC region). In total, 127~129 genes were annotated in the landraces and related *Angelica* species, which comprise 84~85 protein coding genes, 35~36 tRNA genes, and 8 rRNA genes (**Table 3**).

**Table 3:** Characteristics of plastid genomes among the four landraces and two related *Angelica* species.

Species (landraces)	Sample	Genome size (bp)	LSC length (bp)	SSC length (bp)	IR length (bp)	Number of genes	CDS	tRNAs	rRNAs	Total GC (%)	LSC GC (%)	SSC GC (%)	IR GC (%)
Angelica dahurica var. dahurica	KS12	146,871	93,560	17,653	17,829	129	84	36	8	37.53	35.92	31.08	44.95
	AS7	147,076	93,344	17,672	18,030	129	84	36	8	37.54	35.90	31.07	44.97
	BX5	146,890	93,568	17,662	17,830	129	84	36	8	37.53	35.92	31.07	44.95
	CD25	147,081	93,351	17,672	18,029	129	85	36	8	37.54	35.90	31.05	44.97
	HEB18	146,876	93,582	17,668	17,813	129	84	36	8	37.53	35.92	31.07	44.98
‘Hangbaizhi’ landrace	PA26	146,826	93.561	17,629	17,818	127	84	35	8	37.52	35.90	31.07	44.95
	PA29	146,809	93,544	17,629	17,818	129	84	36	8	37.52	35.90	31.07	44.95
‘Chuanbaizhi’ landrace	GY4	146,835	93,570	17,629	17,818	129	85	36	8	37.51	35.89	31.07	44.95
	GY16	146,811	93,546	17,629	17,818	129	84	36	8	37.52	35.90	31.06	44.95
	SN15	146,835	93,570	17,629	17,818	129	84	36	8	37.51	35.89	31.07	44.95
	SN38	146,835	93,570	17,629	17,818	129	84	36	8	37.51	35.89	31.07	44.95
‘Qibaizhi’ landrace	AG29	146,810	93,545	17,629	17,818	127	84	35	8	37.52	35.90	31.07	44.95
	AG37	146,835	93,570	17,629	17,818	129	84	36	8	37.51	35.89	31.07	44.95
‘Yubaizhi’ landrace	CG5	146,811	93,546	17,629	17,818	129	84	36	8	37.52	35.90	31.07	44.95
	CG10	146,811	93,546	17,629	17,818	129	84	36	8	37.52	35.90	31.07	44.95
	YZ12	146,834	93,569	17,629	17,818	129	85	36	8	37.51	35.89	31.07	44.95
	YZ27	146,811	93,546	17,629	17,818	129	84	36	8	37.52	35.90	31.07	44.95
Angelica porphyrocaulis	BJ16	146,894	93,602	17,664	17,814	129	84	36	8	37.53	35.90	31.09	44.98
Angelica dahurica var. formosana	TW01	147,068	93,314	17,598	18,078	129	84	36	8	37.51	35.90	30.95	44.84

**Figure 3:**
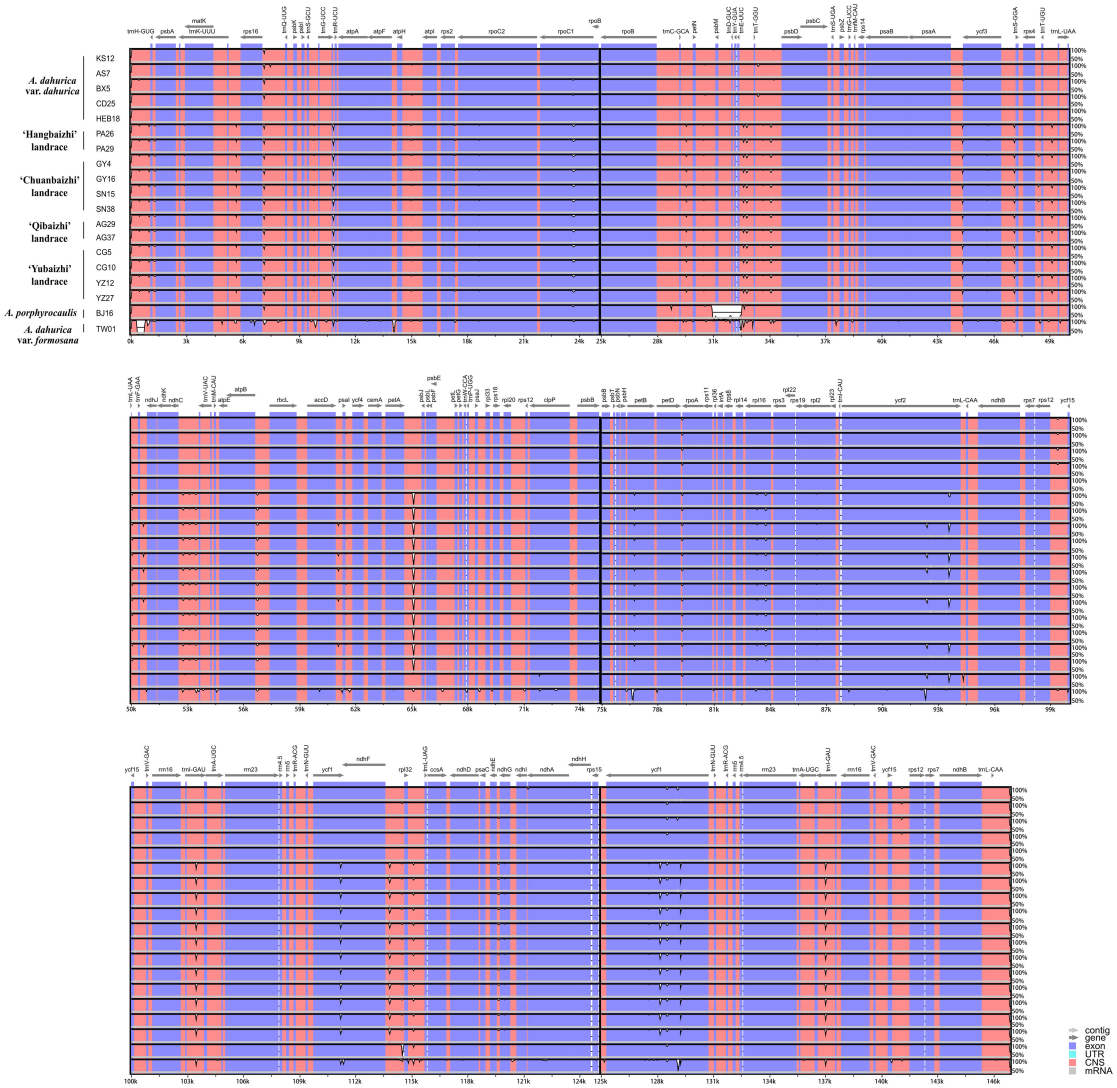
Comparison the plastid genomes of the four landraces and two related *Angelica* species with *A. dahurica* var. *dahurica* (Accession no.: OR209154) as a reference using the Shuffle-LAGAN alignment in mVISTA. The gray arrows and thick black lines above the alignment indicate the genes’ orientations. The Y-axis represents the identity from 50% to 100%.

The divergence among the plastid genomic sequences of the four landraces and two related *Angelica* species was analyzed by mVISTA analysis (**Figure 3**). The results confirmed that the IR region was more conserved than the LSC and SSC regions, and higher variation was found in non-coding regions than that in coding regions. *rpoC1*, *petB*, *rpoA*, *rpl16*, *ycf2*, *trnI-GAU*, and *ycf1* are significantly divergent genes in coding regions, whereas in non-coding regions, highest divergence occurred in some intergenic regions, consisting of *trnL-CAA-trnH-GUG*, *trnK-UUU-rps16*, *rps16-trnQ-UUG*, *trnC-GCA-petN*, *trnE-UUC-trnT-GGU*, *trnT-GGU-psbD*, *psaA-ycf3*, *ycf3-trnS-GGA*, *trnT-UGU-trnL-UAA*, *ndhC-trnV-UAC*, *atpB-rbcL*, *petA-psbJ*, *ndhF-rpl32*, *rpl32-trnL-UAG*, etc.

**Figure 4:**
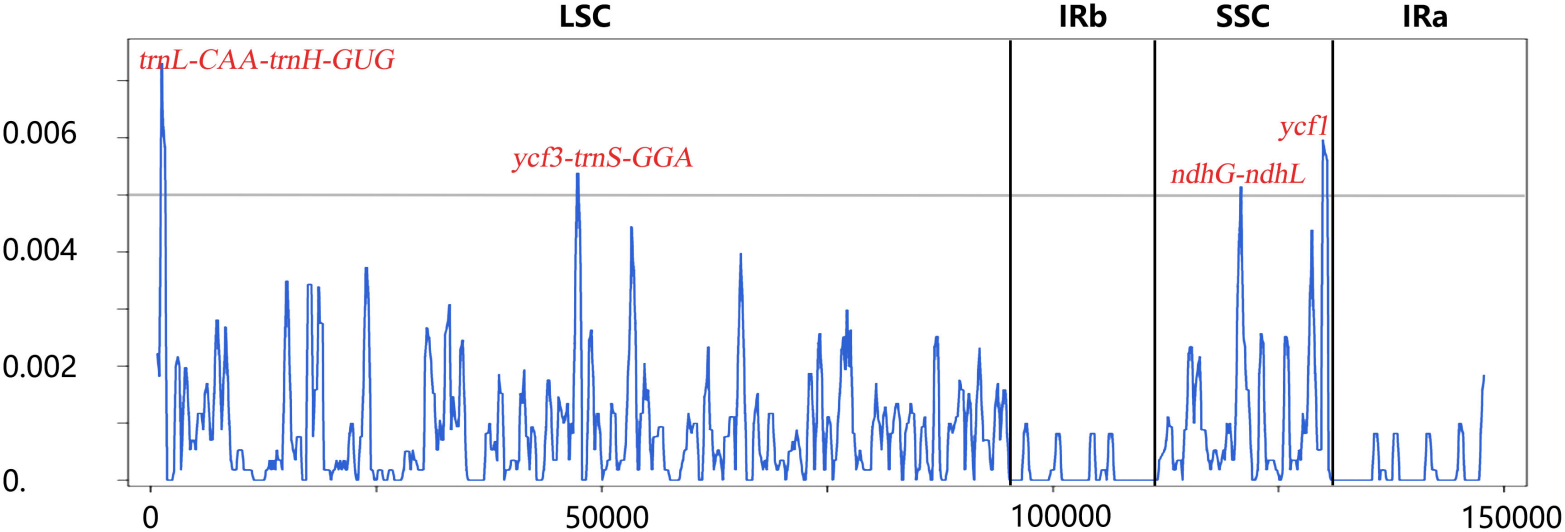
Sliding window analysis based on plastid genomes of the four landraces and two related *Angelica* species. Window length: 600 bp; step size: 200 bp. X-axis: position of the midpoint of a window; Y-axis: nucleotide diversity (Pi) of each window.

Sliding window analysis showed the value of nucleotide diversity (Pi) averaged at 0.00078 ranging from 0 to 0.00731. Four highest mutational hotspots (Pi > 0.005) were detected and marked in **Figure 4**, namely, *trnL-CAA-trnH-GUG* (Pi = 0.00731), *ycf1* (Pi = 0.00596), *ycf3-trnS-GGA* (Pi = 0.00538), and *ndhG-ndhL* (Pi = 0.00515), which could be potentially developed for DNA barcodes in delimiting these related *Angelica* species. In addition, these four hotspots were all located in the LSC and SSC regions, indicating that the IR region was less divergent than the LSC and SSC regions, which was in line with the results of mVISTA analysis. To focus on the variation pattern within four cultivated landraces (12 plastid genomes), mVISTA and sliding window analyses both demonstrated that only few genetic variations existed in intergenic regions (*trnL-CAA-trnH-GUG*, *rps4-trnT-UGU*, etc.) and a coding gene (*ycf2*), and only two mutational sites were detected for *rpl20-rps12* and *ndhF* with extremely low nucleotide diversity (Pi = 0.00051 and Pi = 0.00028, respectively). Consequently, the averaged nucleotide diversity of these landraces was extremely low (averaged Pi = 3.2 × 10^−6^).

### 3.2 Pairwise genetic distance analysis based on ITS and plastid genome

The intraspecific genetic distance within *A. porphyrocaulis* and four landraces was 0.000, whereas that within *A. dahurica* var. *dahurica* was 0.0006, indicating no variation or very limited variation in ITS sequence for these two species and four landraces. Based on the ITS dataset, the maximum interspecific K2P value appeared between *A. dahurica* var. *formosana* and four cultivated landraces (0.043). The minimum interspecific K2P value was found between *A. dahurica* var. *dahurica* and four cultivated landraces (0.005) (**Table 4**). Similarly, the intraspecific genetic distance within *A. dahurica* var. *dahurica* and four landraces were 7.1 × 10^−5^ and 3.2 × 10^−6^, indicating a high consistency within plastid genomes in *A. dahurica* var. *dahurica* and four cultivated landraces, respectively. The maximum interspecific K2P value was identified between *A. dahurica* var. *formosana* and the other two *Angelica* taxa and four landraces (0.003) based on the plastid genomes, whereas the minimum was found to be 0.000 between *A. dahurica* var. *dahurica* and *A. porphyrocaulis* (**Table 4**). These results collectively illustrated that *A. dahurica* var. *dahurica* is very closed with the four landraces of “Baizhi” and *A. porphyrocaulis*.

**Table 4:** Interspecific genetic distance among the four landraces and two related *Angelica* species.

	Angelica porphyrocaulis	Angelica dahurica var. dahurica	Four landraces	Angelica dahurica var. formosana
Angelica porphyrocaulis		0.000	0.001	0.003
Angelica dahurica var. dahurica	0.012		0.001	0.003
Four landraces	0.016	0.005		0.003
Angelica dahurica var. formosana	0.036	0.038	0.043	

Interspecific genetic distances are shown in the lower-left (ITS data) and upper-right (plastid genome data) triangular matrices, respectively.

### 3.3 Phylogenetic and haplotype network analyses

**Figure 5:**
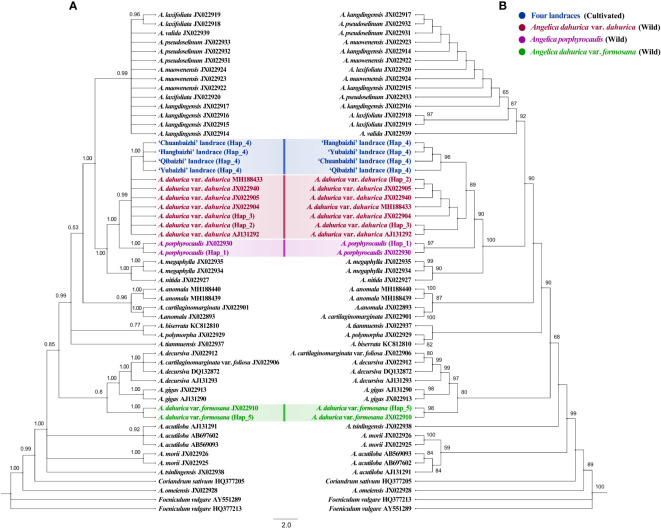
Phylogenetic trees derived from the ITS sequence of 24 *Angelica* species (landraces) with 2 *Foeniculum* species and 1 *Coriandrum sativum* specie as outgroups. **(A)** Bayesian inference (BI) phylogenetic tree with posterior probability (PP) on each nodes. **(B)** Maximum likelihood (ML) tree with bootstrap value (BS) on each branches. Different color circle dots indicate four landraces and two related *Angelica* species. Numbers beside nodes are BS ≥ 50% and PP ≥ 50%.

**Figure 6:**
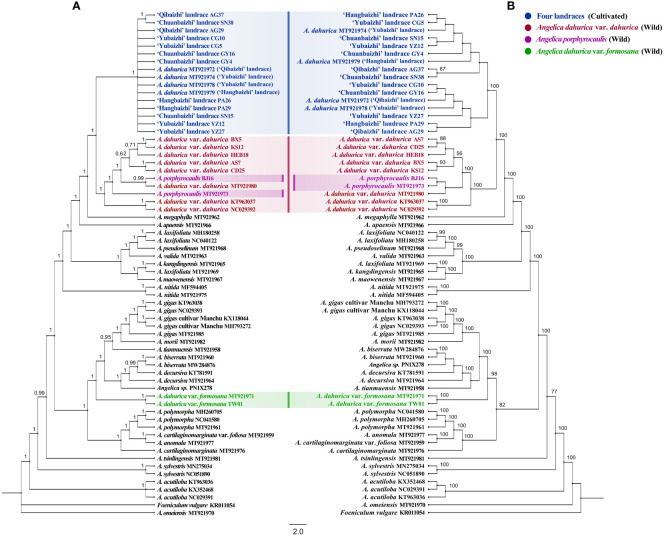
Phylogenetic trees derived from the whole plastid genomes of 30 *Angelica* species (landraces) with 1 *Foeniculum* specie as outgroup. **(A)** Bayesian inference (BI) phylogenetic tree with posterior probability (PP) on each nodes. **(B)** Maximum likelihood (ML) tree with bootstrap value (BS) on each branches. Different color circle dots indicate four landraces and two related *Angelica* species. Numbers beside nodes are BS ≥50% and PP ≥50.

BI and ML analyses of the ITS dataset yielded largely consistent topologies (**Figure 5**), and the same phenomenon was also found in BI and ML analyses of the plastome dataset (**Figure 6**).

In analyses of the ITS dataset (**Figure 5**), the four different landraces of “Baizhi” formed a highly supported lineage (BS = 96%, PP = 1.00) and clustered within the clade including all the individuals of *Angelica dahurica* var. *dahurica*, and this relationship obtained high supports (BS = 89%, PP = 0.99). However, relationships among individuals of both *Angelica dahurica* var. *dahurica* and landraces of “Baizhi” were all resolved as polytomy in BI analysis and weakly supported in ML analysis. The species *A. porphyrocaulis* (including two individuals) was supported as monophyly (BS = 97%, PP = 1.00), and it was sister to the *A. dahurica* var. *dahurica*–”Baizhi” clade with strong supports (BS = 90%, PP = 1.00). In addition, the two individuals of the variety *A. dahurica* var. *formosana* formed a highly supported clade (BS = 98%, PP = 1.00), but they were far isolated from the (*A. dahurica* var. *dahurica*–”Baizhi”)–*A. porphyrocaulis* clade. This variety was sister to the (*A. cartilaginomarginata* var. *foliosa*–*A. decursiva*)–*A. gigas* clade with high support in ML analysis (BS = 80%) but with weak support in BI analysis (PP = 0.80).

In analyses of the plastome dataset (**Figure 6**), all the 16 individuals representing four different landraces of “Baizhi” formed a highly supported clade (BS = 100%, PP = 1.00), which was sister to the *A. dahurica* var. *dahurica*–*A. porphyrocaulis* clade with strong supports (BS = 100%, PP = 1.00), whereas within the strongly supported *A. dahurica* var. *dahurica*–*A. porphyrocaulis* clade (BS = 100%, PP = 1.00), the monophyly of *A. dahurica* var. *dahurica* was not supported, because the species *A. porphyrocaulis* was nested deeply within the former. The species *A. porphyrocaulis* was recovered as polyphyly with strong support in BI analysis (relevant node obtained PP value less than 0.99), although it was recovered as monophyly with weak support in ML analysis. In addition, the far isolated phylogenetic position of *A. dahurica* var. *formosana* from the (*A. dahurica* var. *dahurica*–*A. porphyrocaulis*)–”Baizhi” clade was highly supported, and this variety was sister to a large clade (including species as *A. biserrata*, *A. decursiva*, *A. gigas*, *A. morii*, and *A. tianmuensis*) with strong supports (BS = 98%, PP = 1.00).

Additionally, in the ITS haplotype network (**Figure 1**), haplotype 2 (*A. dahurica* var. *dahurica*) reposed in the center and went through three, four, and eight times mutation, forming the hap 3 (*A. dahurica* var. *dahurica*), hap 4 (four landraces), and hap 1 (*A. porphyrocaulis*), respectively. Therefore, haplotype 2 (*A. dahurica* var. *dahurica*) might be the most primitive haplotype, namely, ancestor haplotype. Haplotype 5 (*A. dahurica* var. *formosana*) experienced 24 times mutation from the ancestor haplotype, indicating a farther relationship between them. The number of mutation times was far greater between haplotypes 5 and 4 than that between haplotype 2 and haplotype 4. Correspondingly, the phylogenetic relationship between *A. dahurica* var. *dahurica* and four landraces was much closer than that between *A. dahurica* var. *formosana* and four landraces.

## 4 Discussion

### 4.1 Low genetic variation within four cultivated landraces indicated one wild progenitor origin

In this study, we evaluated the genetic variation among the four landraces of “Baizhi” based on 86 nrITS sequences from eight populations, and only one haplotype was found for all landraces without any site variations (**Table 2**). Meanwhile, extremely low genetic variation (only two polymorphic sites and Pi = 3.2 × 10^−6^) and K2P genetic distance (3.2 × 10^−6^) occurred within 12 plastid genomes of six populations of four landraces. As the results revealed by this study, we speculated that the four landraces of “Baizhi” might have consistent wild germplasm sources, and they might have originated from single cultivation and domestication, and different landraces had been domesticated during subsequent cultivation processes. This inference was also supported by cytological (Wang et al., 2001b), morphological (Wang et al., 2001a), and molecular evidence (Huang, 2004) supplied in previous studies.

The chromosome karyotypes (2n = 22 = 12m + 2m ^SAT^ +4 sm+ 4st) of four landraces are extremely similar, suggesting no distinction in their cell taxonomy (Wang et al., 2001b). Additionally, Wang et al. (2001a) demonstrated that these four landraces shared common anatomical characteristics of their petals, fruits, and pollen morphology. *Flora of China* morphologically describes that all the landraces have an almost unanimous aerial part of plants, only with slight differences in phenotypes of their roots. Landraces ‘Hangbaizhi’ and ‘Qibaizhi’ have a conical-acuminate shape, with many lenticel-like transverse protrusions slightly arranged in several vertical rows on the gray-brown surface, whereas the roots of landraces ‘Qibaizhi’ and ‘Yubaizhi’ are conical, with surface color of grayish yellow to yellowish brown. The lenticel-like transverse protrusions are irregularly scattered on the root surface (Pan and Watson, 2005). For this medicinal plant that has been domesticated for long, phenotypic differentiation of the medicinal parts between landraces can be explained by environmental adaptations and artificial selections (Korir et al., 2013). Such phenomenon also occurred in many other medicinal plants and crops (Aguirre-Dugua et al., 2013; Hernandez-Teran et al., 2017; Burdejova et al., 2023). Furthermore, the “Baizhi” sourced from landraces ‘Hangbaizhi’ and ‘Chuanbaizhi’ were empirically considered to be much better in quality in market trade as their optimal appearances, which further accelerated the fixation of the high-quality phenotype through continuously artificial selection. In fact, common garden experiments evidenced that there was no significant difference in the root appearances of four landraces under parallel planting conditions (Wang et al., 2001a). Other molecular studies also suggested no significant differentiation among the four landraces (Yuan et al., 2015). Wang et al. (2022) revealed only two mutation sites in *matK* (834 bp) sequences among the landraces. The study also showed the high genetic similarity coefficient (>0.926) for all landraces using SRAP markers. Comprehensively, we thought all of the landraces were derived from only one wild progenitor. Although the monophyly of “Baizhi” (including four different landraces) was strongly supported in the present analyses on the basis of both the ITS dataset (**Figure 5**) and plastome dataset (**Figure 6**), relationships among different landraces have not been well resolved.

### 4.2 The potential wild progenitor of the landraces of “Baizhi”

Wild relatives of cultivated plants are usually considered to be the important resources in increasing yields and developing pests and disease resistance, because the cultivated plants and their non-cultivated relatives usually have similar phylogenetic variations (Maxted et al., 2006; Yao et al., 2016). In recent years, great efforts to clarify the relationship between the cultivated “Baizhi” and wild taxa of the genus *Angelica* have been made, and relevant studies have suggested that this cultivated medicinal plant might be related to three different taxa of *Angelica*, viz. *A. dahurica* var. *dahurica*, *A. dahurica* var. *formosana*, and *A. porphyrocaulis* (Wang et al., 2001a; Huang, 2004; Wang and Jia, 2004; Wang et al., 2020).

The initial argument that *A. dahurica* var. *formosana* was thought to be the wild ancestor of cultivated “Baizhi” obviously is untenable (Zhang, 2012; Wang et al., 2021). As indicated in this study, the variety *A. dahurica* var. *formosana* was nested deeply within a clade that was far isolated from the *A. dahurica* var. *dahurica*–*A. porphyrocaulis*–”Baizhi” clade, which was strongly supported in analyses on the basis of both the ITS dataset (**Figure 5**) and the plastome dataset (**Figure 6**), indicating that *A. dahurica* var. *formosana* should not be the wild progenitor of the cultivated “Baizhi”. Additionally, the isolation of *A. dahurica* var. *formosana* far from the variety *A. dahurica* var. *dahurica* recovered here (**Figures 5**, **6**) also suggested that these two varieties belong to different species, but not a variety of *A. dahurica* as described in *Flora of China* (Pan and Watson, 2005). *A. dahurica* var. *formosana* is endemic to the northern Taiwan of China, which is morphologically characterized by its hairy ovary and fruit, and glabrous lower part of the branches (or with very few hairs) and different from the variant of *A. dahurica* var. *dahurica* (Pan and Watson, 2005). In addition to the mentioned morphological differences, the stem color, diameter of the stem base, and length and width of leaflets of *A. dahurica* var. *formosana* plant are quite different from those of *A. dahurica* var. *dahurica* (Chen and Lu, 2008). Thus, the independent species-level status of *A. dahurica* var. *formosana* should be accepted, and the species name *A. formosana* H. Boissieu should be resurrected.

On the other hand, a very closely related relationship between the variety A. dahurica var. dahurica and the cultivated “Baizhi” was recovered here in analyses on the basis of both the ITS dataset (**Figure 5**) and the plastome dataset (**Figure 6**). In analysis of the ITS dataset, the low phylogenetic resolution among individuals of *A. dahurica* var. *dahurica* indicated that the ITS sequence may have provided a limited phylogenetic signal in disintegrating relationships among these samples. However, these sequences may have provided adequate signal in disintegrating relationships among “Baizhi”, *A. dahurica* var. *dahurica*, and *A. porphyrocaulis*, because the “Baizhi”–*A. dahurica* var. *dahurica* clade and its sister relationship to *A. porphyrocaulis* were all highly supported in BI and ML analyses (**Figure 5**). A highly supported sister relationship between “Baizhi” and *A. dahurica* var. *dahurica* (including *A. porphyrocaulis*) was recovered in analysis of the plastome dataset (**Figure 6**), which seems to be inconsistent to some extent with that derived from analysis of the ITS dataset (**Figure 5**). The haplotype network is widely used for analyzing the relationships within species or among closely related species, which is often more informative than phylogenetic strict consensus trees to display intraspecific DNA sequence variation (Mardulyn, 2012; Garcia et al., 2021; Xiao et al., 2023). Haplotype network analysis in this study gave a good explanation that the landrace haplotype (Hap 4) had only gone through the least steps of mutation from the ancestral haplotype (Hap 2) of *A. dahurica* var. *dahurica* distributed in northern (CD population in Hebei province) and three northeastern provinces (including HEB, DH, BX, AS, and KS populations) of China (**Figure 1**). The nearest K2P genetic distances occurred between the cultivated “Baizhi” and the variety *A. dahurica* var. *dahurica*, further indicating their closely related relationships. A phylogeny among *Angelica* genus and its Apiacae allies generated with ITS and ETS sequences, four cpDNA loci, and morphological data in a previous study (Liao et al, 2013) highly supported one clade of “Baizhi” (Hangbaizhi landrace)–*A. dahurica* var. *Dahurica*. The completely consistent chromosome karyotype (2n=22 = 12m+2msat+4sm+4st) between the variety *A. dahurica* var. *dahurica* and the cultivated “Baizhi” also supported their closed relationship.

It is worth noting that a nuclear-plastid discordance was found for the phylogenetic placement of *A. porphyrocaulis*. In analysis of the ITS dataset, the species was sister to the *A. dahurica* var. *dahurica*–”Baizhi” clade with strong support (**Figure 5**), whereas results from analysis of the plastome dataset showed that the species was nested deeply within *A. dahurica* var. *dahurica* and also with strong supports (**Figure 6**). Considering the highly supported and conflicted placements of *A. porphyrocaulis* in the nuclear tree and plastome tree, we suggest that a possible hybridization event involved in chloroplast capture, which have been reported recently in multiple angiosperm lineages (Liu et al., 2022; Yao et al., 2023), may have occurred between *A. dahurica* var. *dahurica* and *A. porphyrocaulis*. Further morphological evidence and molecular systematics analysis with large-scale sampling and high-resolution gene fragments (such as single-copy nuclear genes) are needed, which would be helpful to elucidate the phylogenetic position of *A. porphyrocaulis*.

Based on the consideration of phylogenetic results and the evolutionary history among *A. dahurica* var. *dahurica*, *A. dahurica* var. *formosana*, *A. porphyrocaulis*, and the cultivated “Baizhi”, we thus suggest that the original variant *A. dahurica* var. *dahurica* seems to be the most plausible wild progenitor of the cultivated “Baizhi”. The geographic origin (wild population of *A. dahurica* var. *dahurica*) and its domestication routes should be further confirmed and clarified with large-scale genomic DNA resequencing data, which will be beneficial for the sustainable utilization and protection of germplasm resources of *A. dahurica* var. *dahurica*.

## 5 Conclusion

In this study, we conducted the genetic variation and phylogenetic analysis on the “Baizhi” landraces (‘Hangbaizhi’, ‘Chuanbaizhi’, ‘Qibaizhi’, and ‘Yubaizhi’) and two *Angelica* species (*A. dahurica* var. *dahurica*, *A. dahurica* var. *formosana*, and *A. porphyrocaulis*), comprising 172 nrITS sequences and 19 chloroplast genomes, aiming to infer the wild original plant of the four landraces and the phylogenetic relationships among the two *Angelica* species. Our findings indicated that the four landraces were originated from the one wild germplasm, and the original variant *A. dahurica* var. *dahurica* is most likely the wild progenitor of the landraces, not the *A. dahurica* var. *formosana* as previous studies reported. Considering phylogenetic inference and evolutionary history, we suggest restoring the independent species-level status of *A. dahurica* var. *formosana*, viz., *Angelica formosana*, and the taxonomic level and phylogenetic position of *A. porphyrocaulis* should be further confirmed. This study preliminarily determined the wild progenitor of “Baizhi” and clarified the phylogenetic relationships among *A. dahurica* var. *dahurica*, *A. dahurica* var. *formosana*, and *A. porphyrocaulis*, which will provide scientific guidance for resources protections and improvement of “Baizhi”.

## Data availability statement

The datasets presented in this study can be found in online repositories. The names of the repository/repositories and accession number(s) can be found in the **Supplementary Material** and below: NCBI (https://www.ncbi.nlm.nih.gov/), The ITS data accessions NO.: OR251501~OR251505, and the plastid genome accessions NO.: OR209144~OR209162.

## Author contributions

YL: Data curation, Formal analysis, Investigation, Methodology, Software, Validation, Visualization, Writing – original draft. GY: Formal analysis, Validation, Writing – review & editing. CH: Formal analysis, Methodology, Validation, Writing – original draft. ZC: Conceptualization, Resources, Writing – review & editing. ET: Conceptualization, Funding acquisition, Supervision, Writing – review & editing.
